# Oxidative stress in preeclampsia and the role of free fetal hemoglobin

**DOI:** 10.3389/fphys.2014.00516

**Published:** 2015-01-13

**Authors:** Stefan R. Hansson, Åsa Nääv, Lena Erlandsson

**Affiliations:** Department of Obstetrics and Gynecology, Institute for Clinical Sciences, Lund UniversityLund, Sweden

**Keywords:** placenta, oxidative stress, fetal hemoglobin, hemolysis, alpha-1-microglobulin

## Abstract

Preeclampsia is a leading cause of pregnancy complications and affects 3–7% of pregnant women. This review summarizes the current knowledge of a new potential etiology of the disease, with a special focus on hemoglobin-induced oxidative stress. Furthermore, we also suggest hemoglobin as a potential target for therapy. Gene and protein profiling studies have shown increased expression and accumulation of free fetal hemoglobin in the preeclamptic placenta. Predominantly due to oxidative damage to the placental barrier, fetal hemoglobin leaks over to the maternal circulation. Free hemoglobin and its metabolites are toxic in several ways; (a) ferrous hemoglobin (Fe^2+^) binds strongly to the vasodilator nitric oxide (NO) and reduces the availability of free NO, which results in vasoconstriction, (b) hemoglobin (Fe^2+^) with bound oxygen spontaneously generates free oxygen radicals, and (c) the heme groups create an inflammatory response by inducing activation of neutrophils and cytokine production. The endogenous protein α1-microglobulin, with radical and heme binding properties, has shown both *ex vivo* and *in vivo* to have the ability to counteract free hemoglobin-induced placental and kidney damage. Oxidative stress in general, and more specifically fetal hemoglobin-induced oxidative stress, could play a key role in the pathology of preeclampsia seen both in the placenta and ultimately in the maternal endothelium.

## Introduction

Preeclampsia is a clinical syndrome that manifests during the second half of pregnancy, afflicting 3–7% of pregnant women. Preeclampsia is one of the leading causes of maternal mortality and morbidity, especially in developing countries (Mackay et al., 2001; Berg et al., 2009). The disease is clinically manifested and defined as *de novo* hypertension with proteinuria after 20 gestational weeks (Redman, 2011). Preeclampsia can develop into a life-threatening condition with general hemolysis, elevated liver enzymes, low platelet counts, and elevated levels of free adult hemoglobin (Hb), classified as the HELLP syndrome (i.e., Hemolysis, Elevated Liver enzymes, Low Platelets) (Schroeder et al., 2002). Preeclampsia can further develop into eclampsia, a severe complication that is defined by the presence of seizures (Mackay et al., 2001). Currently, only symptomatic blood pressure treatment is available for preeclampsia and the only known cure to date is delivery. Hence, preeclampsia causes ~15% of pre-term deliveries. Also, in 25% of cases, preeclampsia leads to intrauterine growth restriction (IUGR) of the fetus. Both pre-term delivery and IUGR result in infant morbidity and substantial health care expenditure (Shmueli et al., 2012). To date there is neither a diagnostic test nor screening tool available for early identification of women at risk of preeclampsia.

Several varying hypotheses and theories regarding the etiology have been put forward over the years. One hypothesis, that was suggested about 25 years ago, is that the extensive vascular endothelial dysfunction in preeclampsia is the result of circulating factors released from the placenta (Rodgers et al., 1988; Roberts et al., 1989). The fact that removal of the placenta is necessary for symptoms to regress has resulted in the theory that the placenta is central in the etiology of preeclampsia (Roberts and Hubel, 2009; Roberts and Escudero, 2012). According to the current theory, preeclampsia evolves in two stages. In brief, the first stage is characterized by a defective placentation, involving incomplete conversion of the spiral arteries by superficial ingrowth of trophoblasts in the decidua (Brosens et al., 2002). As a consequence, uneven blood perfusion, hypoxia, and oxidative stress follow. The second stage of the disease is characterized by the clinical manifestations hypertension, proteinuria, and edema, caused by maternal endothelial damage and systemic inflammation. The endothelial damage is suggested to be caused by placental-derived material or factors (Roberts et al., 1989; Tjoa et al., 2006; Redman and Sargent, 2009).

Despite intense research efforts to unveil the etiology of preeclampsia, it remains enigmatic. However, a growing body of evidence supports the understanding that the disease begins in the utero-placental unit, is amplified by oxidative stress, and ends in the maternal endothelium.

## Oxidative stress

Oxidative stress reflects an imbalance between the formation of oxidative substances and the innate antioxidants that make up the endogenous defense system. Oxidative substances are often free oxygen radicals and peroxides that normally form in small amounts (Buonocore et al., 2010). They can be formed in e.g., the mitochondrial respiratory chain, and when the tissue is exposed to ischemia/reperfusion injury they are produced in larger amounts. Due to their highly reactive properties, they can cause structural and functional damage to cellular DNA, proteins and cell membranes (Tjoa et al., 2006; Valko et al., 2007). In addition to oxidative stress, nitrative stress has also been documented in preeclampsia, which is the covalent modification and nitration of proteins and DNA by peroxynitrite. This occurs in the preeclamptic placenta by the release of reactive oxygen species (ROS) such as superoxide, which interacts with nitric oxide (NO) to form peroxynitrite (Myatt, 2010).

Antioxidants are molecules that inhibit the oxidation caused by oxidative substances, and they are made up of two sub-groups; enzymatic and non-enzymatic antioxidants. Examples of enzymatic antioxidants are superoxide dismutase (SOD), hemoxygenase (HO), and catalase. SOD's enzymatic activity catalyzes the dismutation of superoxide (O^−^_2_) into oxygen and hydrogen peroxide (H_2_O_2_) (McCord and Fridovich, 1988). Thus, SOD is an important antioxidant defense in nearly all cells exposed to oxygen. Three isoforms of HO are known, where HO-1 is inducible in response to oxidative stress and catalyzes the degradation of heme. This produces biliverdin, iron, and carbon monoxide (Kikuchi et al., 2005; Ryter et al., 2006). Catalase is a common enzyme found in nearly all living organisms exposed to oxygen. It catalyzes the decomposition of H_2_O_2_ to water and oxygen (Chelikani et al., 2004).

Examples of non-enzymatic antioxidants are glutathione, thioredoxin, NADH, and NADPH. Glutathione is a key endogenous antioxidant that serves several functions, such as neutralization of free radicals and ROS (Pompella et al., 2003). In addition, glutathione also plays a role in keeping vitamins C and E in their reduced forms (Hughes, 1964; Scholz et al., 1989). Thioredoxin is an oxidoreductase enzyme containing a dithiol-disulfide active site that facilitates the reduction of other proteins by cysteine thiol-disulfide exchange (Holmgren, 1989; Nordberg and Arner, 2001). NADH is a coenzyme found in all living cells, and it has a role in metabolism where it is involved in redox reactions (Belenky et al., 2007). NADPH is, among other things, involved in the protection against ROS by allowing for the regeneration of reduced glutathione (Rush et al., 1985). The NADPH system is also responsible for generating free radicals in neutrophils, which is used in the destruction of pathogens (Ogawa et al., 2008).

α1-microglobulin (A1M) is both an enzymatic and a non-enzymatic antioxidant, present both intra- and extracellularly (Akerstrom and Gram, 2014). It has it's defensive function by working as an antioxidant as well as a binding protein for free radicals and heme (Allhorn et al., 2002; Olsson et al., 2008). A1M is mainly produced in the liver, but can be found in all organs. Once produced, it is deployed to the extracellular fluids and compartments via the blood stream. The synthesis of A1M is up-regulated in response to elevated levels of free radicals, heme, and in particular by free Hb (Olsson et al., 2007). A1M levels are seen to increase in the preeclamptic placenta in an effort by the placenta to up-regulate its defense against increased oxidative stress (Olsson et al., 2010). The protein prevents oxidation and oxidative damage to cells and matrix molecules, and removes heme from cell membranes and cytosol (Olsson et al., 2008).

## Oxidative stress and preeclampsia

In preeclampsia, evidence of oxidative stress can be seen both in the maternal circulation and in the placenta. Placentas from women with preeclampsia have reduced antioxidant capacity compared to normal placentas (Wang and Walsh, 1996; Walsh, 1998). Furthermore, levels of antioxidants in blood from women with preeclampsia have been shown to be reduced, as well as levels of oxidative modifications of proteins and lipoprotein particles (Hubel, 1999; Raijmakers et al., 2004).

Placental trophoblasts and endothelial cells constitute the placental barrier, which effectively separates the fetal and maternal circulation. Oxidative stress and tissue damage is suggested to cause a breach in the barrier and create a leakage of fetal and placenta-derived factors and/or material into the maternal circulation. In stage two of the disease the shedding of placental debris leads to maternal endothelial damage, elevated oxidative stress, and systemic inflammation (Smarason et al., 1993; Knight et al., 1998; Hahn and Holzgreve, 2002; Tjoa et al., 2006). An additional cause to inflammation and vascular damage is the shedding of microparticles from the placenta, induced by placental abnormalities and utero-placental ischemia. The content of the microparticles, such as free Hb and miRNA can further aggravate the systemic oxidative stress (Redman and Sargent, 2008; Tannetta et al., 2013; Cronqvist et al., 2014; Rudov et al., 2014).

Women living at high altitude alter the normal oxygen balance and are shown to have increased risk of developing preeclampsia (Palmer et al., 1999). The effect of oxidative stress on female reproduction has been explored in relation to infertility and assisted reproductive techniques (Agarwal et al., 2012). Pregnancy complications such as recurrent pregnancy loss and spontaneous abortion can develop in response to oxidative stress (Jauniaux et al., 2000; Poston and Raijmakers, 2004). Maternal obesity has also been associated with an increased risk of preeclampsia (Wolf et al., 2001), which could be partly mediated by the increased level of oxidative stress present in obesity (Zavalza-Gomez, 2011). Smoking causes oxidative stress and is an established risk factor for cardio-vascular disease. In pregnant women, smoking causes increased risk of IUGR. However, smoking has also been associated with a protective effect against preeclampsia (Conde-Agudelo et al., 1999). A suggested explanation could be that the observed lower level of sFlt1 in smokers compared to non-smokers is due to the suppression of sFlt1 by carbon monoxide (Karumanchi and Levine, 2010). This is in line with the elevated levels of sFlt1 observed in women with preeclampsia (Levine et al., 2006).

## Oxidative stress causes cellular and systemic effects

### Cellular level

The degree to which the implantation and the conversion of the spiral arteries are deficient has been postulated to influence the degree of oxidative stress and endoplasmic reticulum (ER) stress. Oxidative stress disturbs the normal redox state of the cell, which causes toxic effects on all cellular components, including proteins, lipids, and DNA, and more severe oxidative stress can result in cell death (Valko et al., 2007). During ER stress, unfolded and misfolded proteins accumulate in the ER and activate ER stress-response pathways, also known as the unfolded protein response (Yung et al., 2014). Preeclamptic placentas have been shown to accumulate clusters of misfolded proteins and it is speculated that these aggregates may contribute to the pathophysiology of the disease. The misfolded proteins can also be detected in urine, as a potential biomarker by the Congo red dot test (Buhimschi et al., 2014). ER stress has been shown to be associated with the small placental phenotype in both preeclampsia and IUGR (Burton et al., 2009).

### Systemic level

Inflammation in response to oxidative stress is mediated by the recruitment of leukocytes and the production of pro-inflammatory cytokines, adhesion molecules (ICAM-1 and VCAM-1), and chemokines. This cascade of events is preceded by the presence of heme, which causes production of ROS (Lavrovsky et al., 1994; Markus et al., 2007). Heme has also been shown to induce inflammation via the Toll-like receptors (Belcher et al., 2014).

Endothelial function is affected by the oxidative burden. Superoxide inactivates NO, leading to an increase in peroxynitrite as well as a reduced bioactivity of NO. Furthermore, free Hb can also bind NO (Kim-Shapiro et al., 2006). The depletion of NO results in a series of downstream events, such as impaired endothelium-dependent vasodilation or vasoconstriction, lipoprotein accumulation and oxidative modification, intravascular inflammation and release of pro-inflammatory and vasoactive mediators (Motterlini et al., 1995; Madamanchi et al., 2005). The contents of placental microparticles released into the maternal circulation during preeclampsia also contributes to the elevated level of systemic oxidative stress (Redman and Sargent, 2008; Tannetta et al., 2013; Cronqvist et al., 2014; Rudov et al., 2014).

Primipara women have an increased risk of preeclampsia while pre-conception exposure to paternal antigen gives a reduced risk, implicating an immunological component in the etiology of the syndrome. During the first stage of preeclampsia, maternal adaptation and tolerance to fetal antigen has been suggested to be crucial (Redman and Sargent, 2010).

## Antioxidant protective systems

The human body relies on several antioxidant protective systems. Uric acid is a powerful antioxidant in the human plasma and is a scavenger of free oxygen and radicals. It reduces the oxo-heme oxidant formed by peroxide reaction with Hb and protects erythrocytes from peroxidative damage leading to lysis (Ames et al., 1981). The uric acid level both predicts and correlates with the development of conditions associated with oxidative stress (Sautin and Johnson, 2008) and is suggested to be a marker for oxidative stress (Becker, 1993). Therefore, the concentration of serum uric acid in pregnant women with preeclampsia has been suggested to be associated with disease severity (Pereira et al., 2014).

Vitamins C and E are described as antioxidants against oxidative stress, and their effect on the reduced antioxidant capacity in placentas from women with preeclampsia has been tested in large randomized trials (Poston et al., 2006; Rumbold et al., 2006; Villar et al., 2009; Conde-Agudelo et al., 2011). However, supplementation with vitamins C and E during pregnancy could not be shown to reduce the risk of preeclampsia (Spinnato, 2006).

The compound magnesium sulfate (MgSO_4_) is used as a drug to treat and prevent seizures in severe preeclampsia and eclampsia (Sibai, 2005; Kassie et al., 2014). It has been shown to have a mild vasodilator effect in the maternal circulation (Sontia and Touyz, 2007; Souza et al., 2010) as well as a protective effect on endothelial cells (Kharitonova et al., 2015). It has also been shown to restore cerebral oxygenation impairment seen in patients with severe preeclampsia (Guerci et al., 2014). The exact mechanism of action for MgSO_4_ is not yet understood, but a role as an antioxidant has recently been suggested.

From a clinical perspective it would be of great interest to gain a deeper understanding of the impact of environmental pollutants as well as the influence of nutrients and micronutrients on the oxidative balance in pregnancy in general and preeclampsia in particular (Mistry and Williams, 2011). Exposure to air pollution, especially to ultra-fine particles has been shown to induce oxidative stress (Terzano et al., 2010). Furthermore, a positive association between preeclampsia and exposure to air pollutants, such as nitrogen oxides, nitrogen monoxide, nitrogen dioxide, carbon monoxide, ozone, particulate matter of <2.5 μm and particulate matter of <10 μm, has been shown in recent studies (Wu et al., 2009, 2011; Lee et al., 2013; Malmqvist et al., 2013).

Deficiency of micronutrients such as selenium, copper (Cu), zinc (Zn), and manganese (Mn) during pregnancy can affect antioxidant capacities in the mother and contribute to poor pregnancy outcomes e.g., preeclampsia (Rumbold et al., 2008). The antioxidant defenses found in placenta includes the selenium-dependent glutathione peroxidases, thioredoxin reductases, selenoprotein-P, and Cu/Zn and Mn SOD's (Wang and Walsh, 1996; Vanderlelie et al., 2005; Rayman, 2009). Studies have shown a correlation between low serum selenium concentrations and reduced antioxidant function in preeclampsia, suggesting that selenium status is important. This is further supported by placebo-controlled randomized controls trials where pregnant women were given selenium supplementation, resulting in lower incidence of pregnancy-induced hypertension and/or preeclampsia (Han and Zhou, 1994; Rumiris et al., 2006). Cu is an essential cofactor for several important enzymes including catalase and SOD (Gambling et al., 2008). Cu is a redox-active transition metal with pro-oxidant properties and can catalyze the formation of free radicals, thereby inducing oxidative stress in preeclampsia (Serdar et al., 2006). However, when Cu is associated in the Cu/Zn SOD it functions as an antioxidant expressed in both maternal and fetal tissue (Ali Akbar et al., 1998). Studies have shown increased levels of Cu in placenta, maternal serum, and amniotic fluid in preeclamptic women compared to controls (Brophy et al., 1985; Dawson et al., 1999; Serdar et al., 2006). However, data on Cu status throughout normal pregnancies as well as early preeclamptic pregnancies is inadequate, and it remains unclear whether Cu deficiency is a public health problem (Arredondo and Nunez, 2005).

Zn has antioxidant functions through the Cu/Zn SOD (Izquierdo Alvarez et al., 2007). Zn supplementation studies show reduced incidence of pregnancy induced hypertension (Goldenberg et al., 1995), and Zn deficiency has been associated with preeclampsia with decreased levels in placental tissue and maternal serum (Brophy et al., 1985; Dawson et al., 1999; Kumru et al., 2003). Mn is also a cofactor for important enzymes, such as the antioxidant Mn SOD which is involved in protection against oxidative stress in the placenta (Ademuyiwa et al., 2007). Studies have indicated that whole blood Mn concentrations are lower in women with fetal growth restriction (Vigeh et al., 2008). Furthermore, umbilical cord whole blood in preeclampsia has been shown to have reduced levels of Mn (Jones et al., 2010).

Dietary nitrate, in e.g., beetroot juice, is converted *in vivo* to bioactive nitrogen oxides such as NO and may be used as nitrate supplementation (Lundberg et al., 2008). It has been shown to reduce blood pressure, increase blood flow, and modulate oxidative stress (Webb et al., 2008; Carlstrom et al., 2011; Lundberg et al., 2011). Beetroot juice has recently been shown to improve placental vascular function during pregnancy in mice (Cottrell et al., 2014).

## Fetal hemoglobin in the pathogenesis of preeclampsia

**Figure 1:**
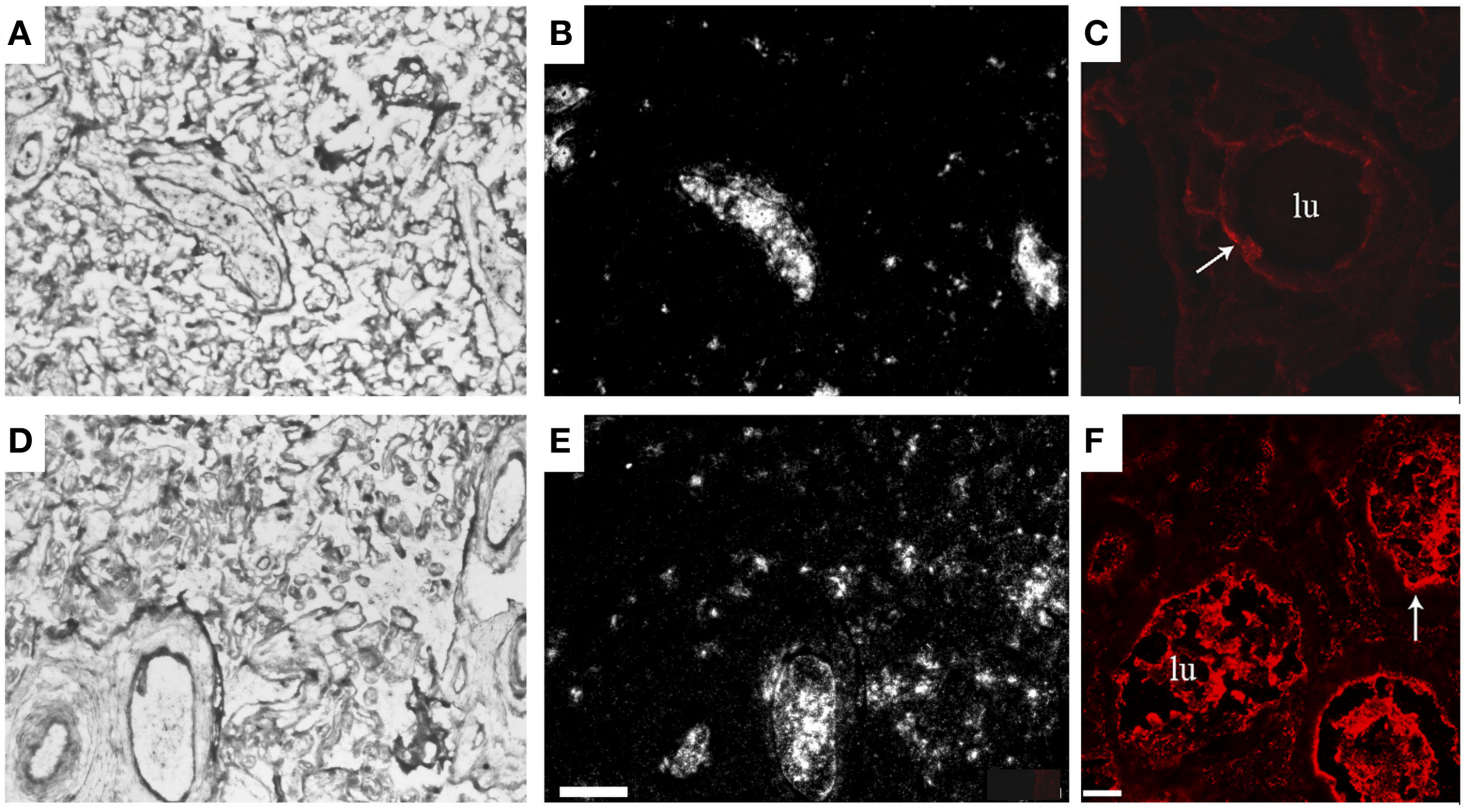
**Increased placental expression of HbF in preeclampsia**. Representative images from *in situ* hybridizations of human placenta, displaying the villous section of the placenta. HbF mRNA expression (α-chain) in a control placenta shown as light field image **(A)** and as dark field image **(B)**, and in a preeclamptic placenta shown as light field image **(D)** and as dark field image **(E)**. HbF expression was especially seen in and around blood vessels, with several scattered cells detected in the villous section of the preeclamptic placenta. HbF protein expression (γ-chain) is shown with a red fluorescent marker in control placenta **(C)** and in preeclamptic placenta **(F)**. In the preeclamptic placenta there is a strong expression of HbF in the vascular lumen (lu), but HbF is also expressed in the vascular endothelium (arrow) as well as in the extravascular section of the villous stroma. The placenta from control shows no expression of HbF in the vascular lumen (lu), but expression is detected in the vascular endothelium (arrow). Scale bars for **(A,B,D,E)** = 100 μm and for **(C,F)** = 25 μm. Modified from Centlow et al. (2008).

With the use of microarray and proteomics technologies we studied gene expression in placentas from normal patients and patients with preeclampsia. Through the use of a subtraction library, a total of 750 preeclampsia-associated genes were identified, which were used to manufacture analytical chips for microarray analysis (Centlow et al., 2008). Increased expression of genes involved in inflammation, apoptosis, and oxidative stress were found in the preeclamptic placentas. More specifically, an increased expression of fetal Hb (HbF), seen as both elevated mRNA and accumulation of the protein, was reported (Figure 1). *In situ* hybridization and immunohistochemistry were used to localize the cells that expressed HbF in the placenta, and these were identified as hematopoietic stem cells located to the vascular lumen (Figure 1).

## Free hemoglobin causes oxidative stress

Hb is composed of four globin chains, each with an embedded iron-containing heme group with a high affinity to oxygen. The most common variants of the globin chains are α, β, and γ, and that are used differently during normal embryonic and fetal development. HbF consists of two α- and two γ-chains and is the main Hb form in fetuses, being expressed from approximately gestational week 10 to birth. HbF comprises <1% of the Hb in adults. Adult Hb (HbA) consists of two α- and two β-chains, and makes up >95% of the Hb after 6 months of age.

The redox activity of the iron atom in the heme group serves as the basis for the strong oxidative reactivity of free Hb. Free Hb is defined as extracellular unbound Hb. During hemolysis, erythrocytes rupture and free Hb leaks out into the circulation. Eryptosis is a suicidal erythrocyte death, stimulated by oxidative stress, energy depletion, calcium imbalance, and a wide range of xenobiotic compounds. It is inhibited by erythropoietin and NO (Lang et al., 2012). Eryptosis affects the capillary beds by fostering thrombosis. It is seen in several systemic conditions such as diabetes, renal insufficiency and malaria, conditions well-known as risk factors for preeclampsia.

The vasoconstrictive effect of free Hb is a result of the ferrous Hb (Fe^2+^) binding strongly to the vasodilator NO, thereby reducing the availability of NO. Currently, NO donors are being investigated in clinical trials as therapeutics for IUGR and preeclampsia (Cindrova-Davies, 2014). Hb (Fe^2+^) with bound oxygen (OxyHb) spontaneously generates free oxygen radicals. This results in aggregated and oxidized forms of the molecule, degradation products, and free iron and heme groups. As a result, Hb and its degradation products are toxic and can cause oxidative stress, hemolysis, vasoconstriction, kidney, and vascular endothelial damage (Buehler and D'Agnillo, 2010). Heme groups have a direct effect on the inflammatory response by inducing activation of neutrophils and induction of cytokine production (Kumar and Bandyopadhyay, 2005).

Several defense mechanisms exist to protect against the harmful effects of free Hb. Antioxidants such as vitamins C and E protect against oxidative stress. The blood plasma scavenger proteins haptoglobin and hemopexin bind free Hb and free heme groups, respectively, and the formed complexes are eliminated from the blood by cellular uptake via the two receptor-mediated pathways CD163 on macrophages and CD91 in the liver (Kristiansen et al., 2001; Ascenzi et al., 2005). In fact, haptoglobin levels are reduced in preeclampsia, indicating a depletion of the protective systems against free Hb (Olsson et al., 2010).

## Free hemoglobin causes placental damage

**Figure 2:**
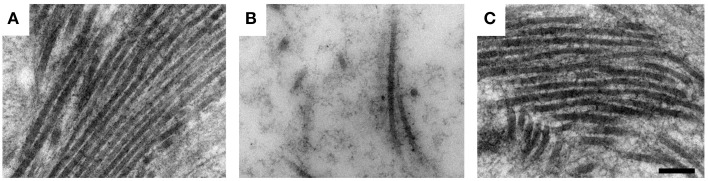
**Free Hb causes placental damage *ex vivo* which can be ameliorated by A1M**. Transmission electron microscopy analysis of *ex vivo* perfused human placenta. **(A)** Non-perfused human placenta. **(B)** Free Hb was added to the fetal circulation and caused severe damage to the extracellular matrix with an almost complete elimination of the collagen fibers. **(C)** A1M was added to the maternal circulation at the same time as Hb was added to the fetal circulation, and prevented the damaging effects of Hb on the extracellular matrix, displaying normal collagen fibers. Scale bars: 200 μm. Adapted from May et al. (2011).

**Figure 3:**
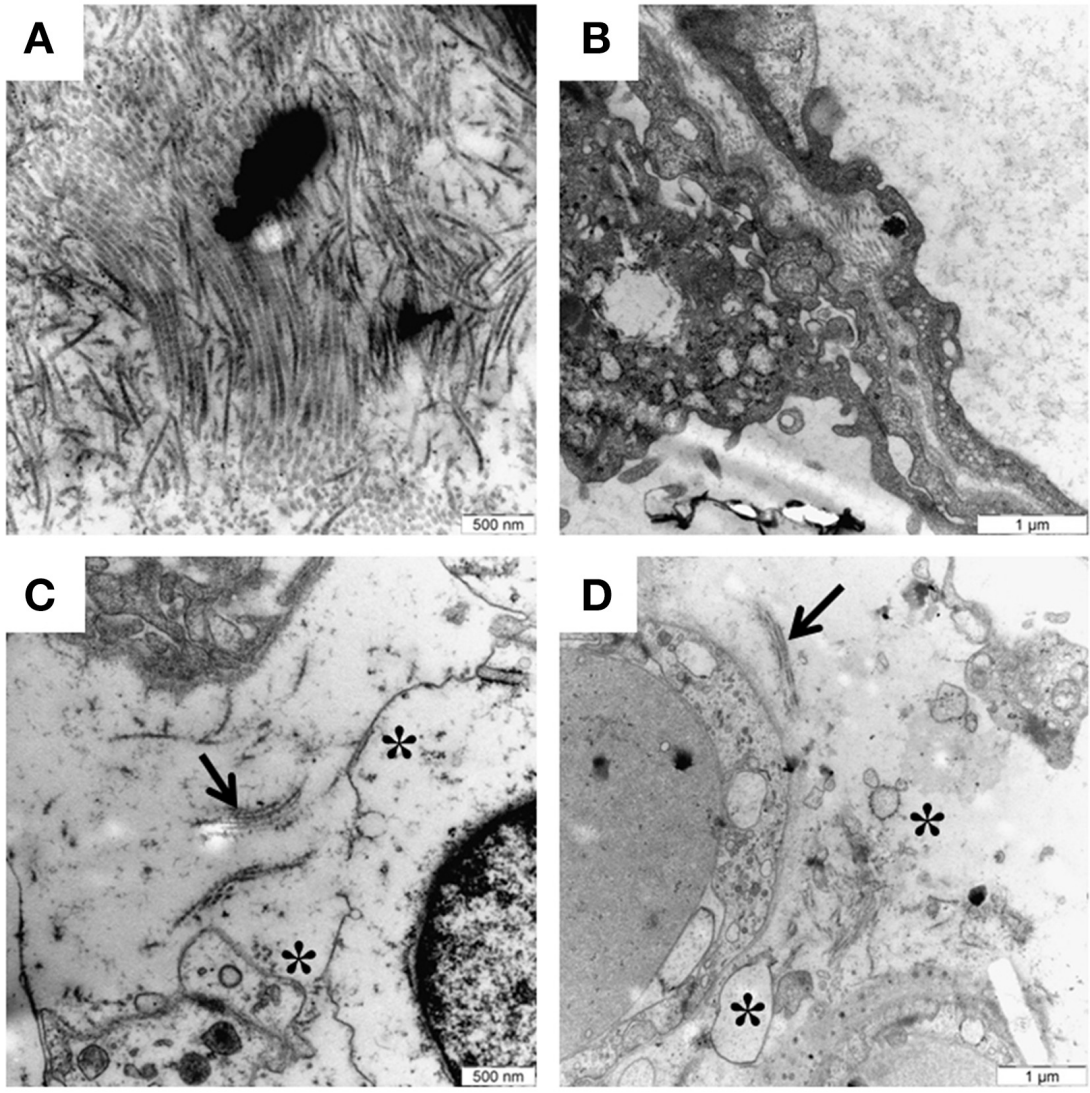
**Free HbF causes structural and cellular damage to placenta tissue *in vivo***. Transmission electron microscopy analysis of placenta tissue from pregnant rabbits infused with free HbF. **(A,B)** Normal placental tissue from control animals showing extracellular matrix with dense bundles of collagen fibers. **(C,D)** HbF causes severe damage to the extracellular matrix with loss of collagen fibers (indicated by arrows), extracellular apoptotic bodies, cell debris and a lot of empty extracellular space (indicated by stars). Scale bars: **(A,C)** = 500 nm; **(B,D)** = 1 μm.

Recently our group has shown that free Hb is a potential key factor in the pathogenesis of preeclampsia. By aggravating oxidative stress, causing damage to the placental barrier and leaking into the maternal bloodstream, it causes endothelial damage and vasoconstriction (Centlow et al., 2008; May et al., 2011). To study the toxic effect of free Hb on placental tissue, the placenta perfusion model was used. This well-documented *ex vivo* system mimics the pregnant *in vivo* situation in humans in a way not possible by using placenta cell culture or animal models (Schneider and Huch, 1985; Di Santo et al., 2007). In this system, free Hb was added to the fetal circulation. A preeclampsia-like situation developed within 10 min, displaying a steep increase in blood pressure. Signs of placental barrier breach appeared after ~1 h of perfusion, with leakage of nutrients from the fetal circulation into the maternal circulation. Transmission electron microscopy (TEM) confirmed the tissue damage with considerable damage to the extracellular matrix, with an almost complete elimination of the collagen fibrils that maintain the tissue structure (Figure 2). Further, there were widespread cellular changes with damage to membranes, nuclei and mitochondria, and formation of apoptotic vesicles and vacuoles. Gene profiling of placentas perfused with free Hb showed a similar genetic profile as women with preeclampsia. These *ex vivo* findings suggest that free Hb plays an important role in the disease etiology (Centlow et al., 2009; May et al., 2011). To further study the effects of free HbF, we established a pregnant rabbit preeclampsia model where pregnant rabbits were infused with species-specific free HbF, starting at mid-gestation until term. TEM analysis of the placenta revealed structural damages with a dramatic reduction of the collagen fibers in the extracellular matrix, as well as cellular damages such as mitochondrial swelling, aberrant intracellular nuclear membranes, damaged electron dense barrier, and high levels of apoptotic bodies (Figure 3). In the pregnant ewe preeclampsia model, starvation induces preeclampsia-like symptoms by causing hemolysis with subsequent release of free Hb (Wester-Rosenlof et al., 2014). Also in this model TEM analysis could confirm tissue damage in placentas from starved ewes, with an almost complete elimination of the collagen fibers as well as cellular damages (Wester-Rosenlof et al., 2014).

## Free hemoglobin causes kidney damage

The proteinuria in preeclampsia is associated with glomerular injuries in the kidney, known as glomerular endotheliosis. Endotheliosis includes swelling of endothelial cells and loss of endothelial fenestration, as well as occlusion of capillary lumens in some cases (Lafayette et al., 1998; Stillman and Karumanchi, 2007). Podocytes form the slit diaphragm, which is crucial for maintaining the size-selective nature of the glomerular filtration barrier in the kidney, and alterations in the podocytes have been associated with proteinuria in preeclampsia (Garovic et al., 2007; Henao et al., 2008; Henao and Saleem, 2013). This indicates that podocytes are a crucial part in explaining the loss of filtration capacity in the preeclamptic kidneys. Podocyturia has also been associated with preeclampsia (Garovic et al., 2007). Glomerular endotheliosis has long been considered pathognomonic for preeclampsia (Stillman and Karumanchi, 2007), however, these changes have also been observed, albeit to a lesser extent, by pregnancy-induced hypertension (absence of proteinuria) and in normal pregnancy (Strevens et al., 2003). Furthermore, changes to the podocytes, and their interaction with the glomerular endothelium has recently been shown to be important in the onset of proteinuria and podocyturia (Wagner et al., 2012; Craici et al., 2013). In a mouse knockout model where podocytes lack VEGF, endotheliosis, and proteinuria were seen (Eremina et al., 2003). The capillary leakage in the glomeruli contributes to the observed proteinuria. It is further shown that the renin-angiotensin system (RAS) plays a role in the development of hypertension by an increased vascular response to angiotensin II (Wang et al., 2009; Rodriguez et al., 2012). Autoantibodies against the angiotensin II AT1 receptor has been shown in preeclampsia (Dechend et al., 2005; Karumanchi and Lindheimer, 2008).

In severe forms of preeclampsia/HELLP/eclampsia, the damaged endothelium activates platelets and the coagulation system, which may cause sub-acute/acute disseminated intravascular coagulation (DIC) (Thachil and Toh, 2009). In DIC, there is a dysregulation of coagulation and fibrinolysis causing decreased platelet count and fibrinogen levels, and increased consumption of antithrombin. Especially in the kidneys and the liver, a gradual fibrin formation occurs.

**Figure 4:**
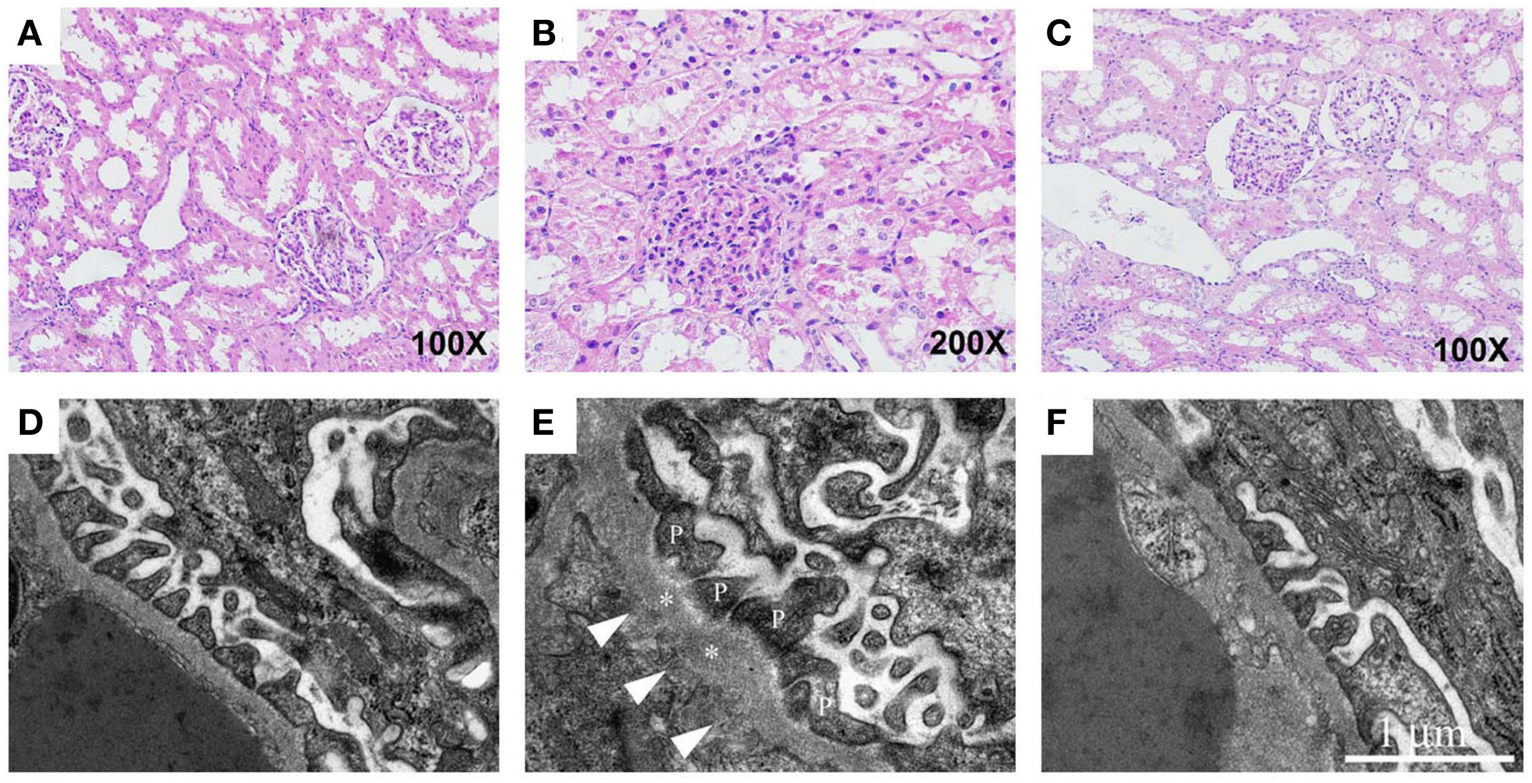
**Free Hb causes kidney damage in pregnant ewes which can be ameliorated by A1M**. The renal tissue from starved pregnant ewes was studied by light microscopy on cortical specimens stained with hematoxylin and eosin **(A–C)** and by transmission electron microscopy **(D–F)**. **(A)** Normal cortical tissue morphology in control ewes. The renal tubules show minor signs of postmortem changes but the height of the epithelium is normal, and only discrete cytoplasmic vacuolization's are seen. The glomeruli demonstrate open capillary loops and no signs of segmentation. **(B)** Clear signs of glomerular endothelial swelling, seen as closure of capillary loops, and non-isometric vacuolization of the tubular epithelium in the starved ewes. **(C)** In the A1M-treated starved ewes, only small tubular changes compatible with acute tubular necrosis are present and to a milder degree than seen in starved ewes. Also, no signs of glomerular endothelial swelling can be seen. **(D)** Normal ultrastructure of the glomerular area in control ewes. **(E)** Disturbed morphology of the podocytes in the starved animals. The arrows point at abnormal regions on the basement membrane with fenestrations underneath. The asterisks mark the basal membrane and P indicates the podocyte foot extensions. **(F)** Normalized ultrastructure was observed after A1M treatment. Magnification: **(A,C)** 100×; **(B)** 200×. Scale bars: **(D–F)** 1 μm. Adapted from Wester-Rosenlof et al. (2014).

Patients with preeclampsia exhibit a reduced glomerular filtration rate. Behind this lies a partly reduced renal blood flow and a reduced filtration area caused by reduced fenestration in the glomerular endothelium and fibrin deposits. In the HELLP syndrome, acute kidney injury is seen in approximately 3–15% of cases (Fakhouri et al., 2012), where glomerular endotheliosis and acute tubular necrosis are typical findings. The acute high level of free Hb is likely to contribute to the kidney damage. To study the effect of free HbF on the function of a normal rat kidney *in vivo*, free HbF was systemically infused into rats and the permeability of the glomerular filtration barrier in the kidney was studied (Sverrisson et al., 2014). Our findings demonstrate that free HbF induces an increase in the glomerular permeability to macromolecules through the induction of oxidative stress. This change is believed to occur via a direct interaction between HbF and the endothelium via ROS and/or NO (Sverrisson et al., 2014). Also, in the pregnant ewe preeclampsia model, free Hb induced an increase in the glomerular permeability as well as typical glomerular endotheliosis (Wester-Rosenlof et al., 2014) (Figure 4). Other hemolytic diseases such as autoimmune hemolytic anemia, sickle cell anemia and malaria are also known to cause kidney disease (Bunn et al., 1977).

## A1M counteracts tissue damage caused by free Hb

In several recent studies our group has evaluated the protective effect and the therapeutic potential of A1M. Using the *ex vivo* placenta perfusion model described above (May et al., 2011), preeclampsia-like symptoms developed shortly after perfusion of free Hb to the fetal circulation. To evaluate the potential therapeutic effect, A1M was added to the maternal circulation of the perfused placenta in an attempt to mimic a potential future intravenous therapy. A1M reversed the damages caused by free Hb, where the Hb leakage from the fetal to the maternal circulation ceased completely, and no structural damages to the placental tissue were detected (Figure 2). Gene profiling showed that genes coding for the structural components of the extracellular matrix were up-regulated, suggesting that a healing process was initiated by A1M.

The role of A1M was further studied in two different *in vivo* animal models. In the first study, systemically infused free HbF in rats caused an increase in the glomerular permeability to macromolecules in the kidney through the induction of oxidative stress. This was counteracted by A1M and resulted in a restored glomerular filtration barrier (Sverrisson et al., 2014). The pregnant ewe preeclampsia model was also used to evaluate the therapeutic potential of A1M (Wester-Rosenlof et al., 2014). In this model, free Hb induced preeclampsia-like symptoms and intravenous infusion with A1M ameliorated the structural tissue damages seen in both kidney (Figure 4) and placenta, as well as restored the glomerular filtration rate in the kidney.

## Other contributory factors to the development of preeclampsia

The maternal injured or activated endothelium in preeclampsia has been shown to release several factors including endothelin-1, fibronectin, von Willebrand factor, thrombomodulin, markers of oxidative stress and inflammatory cytokines (Musci et al., 1988; Thorp et al., 1990; Nova et al., 1991; Taylor et al., 1991; Hsu et al., 1993; Minakami et al., 1993; Deng et al., 1994; Cackovic et al., 2008). Other factors with vasodilation properties, e.g., NO and prostacyclin have been reported to be reduced in preeclampsia while factors such as angiotensin II and thromboxane have been shown to be elevated. This imbalance between vasodilation and vasoconstriction factors contributes to increased vasoconstriction and hypertension (Lowe, 2000; Ariza et al., 2007).

Endothelial damage also triggers the activation of the coagulation system. Increased thrombocyte activity is seen in preeclampsia, which in severe cases can lead to DIC (Thachil and Toh, 2009). Thrombosis in the placental vessels further impairs vascular perfusion, leading to a negative spiral of illness. In addition, the leaking fetal and placental factors are usually foreign to the maternal immune system, further contributing to the inflammation that aggravates the endothelial damage (Rusterholz et al., 2007; Redman and Sargent, 2009; Messerli et al., 2010).

Signs of chronic inflammation, increased dendritic cell numbers and macrophage infiltration are seen in the preeclamptic placenta, indicating a pathological immune response (Salafia et al., 1995; Hiby et al., 2004; Lockwood et al., 2006; Huang et al., 2008). Paternal antigen exposure has been shown to be a risk factor for preeclampsia. In addition, women with an incomplete or disturbed immune system (e.g., untreated HIV positive women) are less likely to develop preeclampsia, further suggesting an immunological dysfunction to be part of the etiology of preeclampsia (Wimalasundera et al., 2002).

Another active area of investigation focuses on the role of angiogenic factors such as sFlt1, VEGF, PlGF, TGFβ, and sEng in preeclampsia. The maintenance of a healthy vascular endothelium relies on a proper balance between proangiogenic and antiangiogenic factors released during pregnancy. Discordance in these has been postulated to be a possible cause of endothelial dysfunction seen in preeclampsia (Kendall and Thomas, 1993; Kendall et al., 1996; Clark et al., 1998; Wimalasundera et al., 2002; Levine et al., 2006; Sela et al., 2008; Heydarian et al., 2009; Thomas et al., 2009; Walshe et al., 2009).

## Concluding remarks

The prevailing theory is that preeclampsia is a syndrome that starts in the placenta and ends in the maternal endothelium. As reviewed here, oxidative stress in general, and more specifically oxidative stress induced by free HbF, could play a key role in the pathological mechanisms in both the placenta and ultimately the maternal endothelium as seen in preeclampsia. Gene and protein profiling studies have shown that free HbF forms and accumulates in the placenta in preeclampsia.

Oxidative stress appears to hold a central position during stage one of preeclampsia, and contributes to the clinical manifestations during stage two of the syndrome. Oxidative stress is amplified in a vicious circle of overproduction and release of Hb, free HbF in preeclampsia and free HbA in the HELLP syndrome. The maternal constitutional factors can either predispose or protect against the increased oxidative stress. Depending on the individual constitutional factors, the same oxidative insult may give rise to several manifestations and forms of preeclampsia, as well as varying severity.

The endogenous A1M protein, with radical and heme binding properties, has shown both *ex vivo* and *in vivo* to have the ability to counteract free Hb-induced placental and kidney damage, and to restore both placental barrier and glomerular filtration barrier functions.

### Conflict of interest statement

Stefan R. Hansson holds patents for the diagnosis and treatment of preeclampsia. Stefan R. Hansson is co-founder of the companies Preelumina Diagnostics AB and A1M Pharma AB. The other authors declare that the research was conducted in the absence of any commercial or financial relationships that could be construed as a potential conflict of interest.

## Abbreviations

A1M: alpha-1-microglobulin
IUGR: intrauterine growth restriction
HELLP: hemolysis, elevated liver enzymes, and low platelets syndrome
SOD: superoxide dismutase
HO: hemoxygenase
O^−^_2_: superoxide
H_2_O_2_: hydrogen peroxide
ROS: reactive oxygen species
ER: endoplasmatic reticulum
NO: nitric oxide
sFlt1: soluble fms-like tyrosine kinase 1
VEGF: vascular endothelial growth factor
PlGF: placental growth factor
TGFβ: transforming growth factor-beta
sEng: soluble endoglin
RAS: renin-angiotensin system
DIC: disseminated intravascular coagulation.
